# (*Z*)-3α-(1,3-Dioxoisoindolin-2-yl)-17(20)-pregnene

**DOI:** 10.1107/S1600536811027632

**Published:** 2011-07-16

**Authors:** Yue Qi, Nan Qin, Hong-Quan Duan

**Affiliations:** a School of Pharmaceutical Sciences, Research Center of Basic Medical Scienses, Tianjin Medical University, Tianjin 300070, People’s Republic of China

## Abstract

The title compound, C_29_H_37_NO_2_, crystallized with two independent mol­ecules in an asymmetric unit in which the conformation of the cyclo­hexyl ring of the pregnene moiety bonded to the 3α-(1,3-dioxoisoindolin-2-yl)- ring system differs: in one mol­ecule it is in a chair conformation, while in the other it exhibits a half-chair conformation. The other six-membered rings in the pregnene moiety are in chair conformations and the five-membered rings are in envelope forms in both mol­ecules. In both mol­ecules, the 3α-(1,3-dioxoisoindolin-2-yl)- ring systems are individually approximately planar, with r.m.s. devtaions 0.0148 and 0.0264 Å. The structure is consolidated by inter­molecular C—H⋯O hydrogen-bonding inter­actions involving the carbonyl O atoms and methyl, methyl­ene and methyl­idyne groups, resulting in a two-dimensional structure.

## Related literature

The title compound was synthesized from epiandrosterone, a pregnane alkaloid isolated from *Pachysandra axillaris*, a traditional chinese medicine. For the biological activity of *Pachysandra axillaris*, see: Sun *et al.* (2010 ▶). For the synthesis of the title compound, see: Batcho *et al.* (1981 ▶). For a related structure, see: Ishida *et al.* (1981 ▶). For the absolute structure, see: Pollard & Ahmed (1971 ▶). 
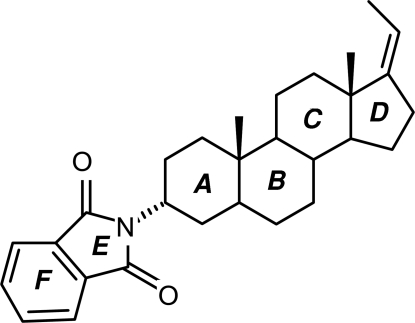


## Experimental

### 

#### Crystal data

C_29_H_37_NO_2_
*M* *_r_* = 431.60
Monoclinic, 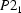

*a* = 7.5895 (10) Å
*b* = 31.355 (3) Å
*c* = 9.8912 (12) Å
β = 93.056 (6)°
*V* = 2350.4 (5) Å^3^
*Z* = 4
Mo *K*α radiation
μ = 0.08 mm^−1^
*T* = 113 K
0.22 × 0.20 × 0.12 mm

#### Data collection

Rigaku Saturn724 CCD diffractometer
Absorption correction: multi-scan (*CrystalClear*; Rigaku/MSC, 2005 ▶) *T*_min_ = 0.984, *T*_max_ = 0.991
17383 measured reflections
5642 independent reflections
5296 reflections with *I* > 2σ(*I*)
*R*_int_ = 0.052

#### Refinement

*R*[*F* ^2^ > 2σ(*F* ^2^)] = 0.039
*wR*(*F* ^2^) = 0.096
*S* = 1.04
5642 reflections
583 parameters
1 restraint
H-atom parameters constrained
Δρ_max_ = 0.26 e Å^−3^
Δρ_min_ = −0.27 e Å^−3^

### 

Data collection: *CrystalClear* (Rigaku/MSC, 2005 ▶); cell refinement: *CrystalClear*; data reduction: *CrystalClear*; program(s) used to solve structure: *SHELXS97* (Sheldrick, 2008 ▶); program(s) used to refine structure: *SHELXL97* (Sheldrick, 2008 ▶); molecular graphics: *SHELXTL* (Sheldrick, 2008 ▶); software used to prepare material for publication: *CrystalStructure* (Rigaku/MSC, 2005 ▶).

## Supplementary Material

Crystal structure: contains datablock(s) I, global. DOI: 10.1107/S1600536811027632/pv2421sup1.cif

Structure factors: contains datablock(s) I. DOI: 10.1107/S1600536811027632/pv2421Isup2.hkl

Supplementary material file. DOI: 10.1107/S1600536811027632/pv2421Isup3.cdx

Supplementary material file. DOI: 10.1107/S1600536811027632/pv2421Isup4.cdx

Supplementary material file. DOI: 10.1107/S1600536811027632/pv2421Isup5.cdx

Additional supplementary materials:  crystallographic information; 3D view; checkCIF report

## Figures and Tables

**Table 1:** Hydrogen-bond geometry (Å, °)

*D*—H⋯*A*	*D*—H	H⋯*A*	*D*⋯*A*	*D*—H⋯*A*
C1—H1*B*⋯O2	0.99	2.25	3.027 (3)	134
C5—H5⋯O4^i^	1.00	2.59	3.237 (3)	122
C21—H21*C*⋯O3^ii^	0.98	2.60	3.501 (3)	153
C31—H31*A*⋯O2^iii^	0.99	2.47	3.361 (3)	150

Symmetry codes: (i) 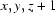; (ii) 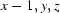; (iii) 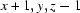.

**Fig. 1.**
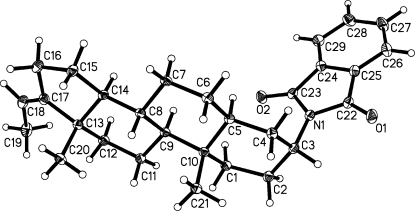
One independent molecule in the asymmetric unit of the title compound, with 30% probability displacement ellipsoids.

**Fig. 2.**
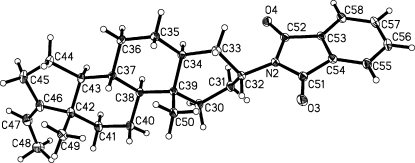
The other independent molecule in the asymmetric unit of the title compound, with 30% probability displacement ellipsoids.

**Fig. 3.**
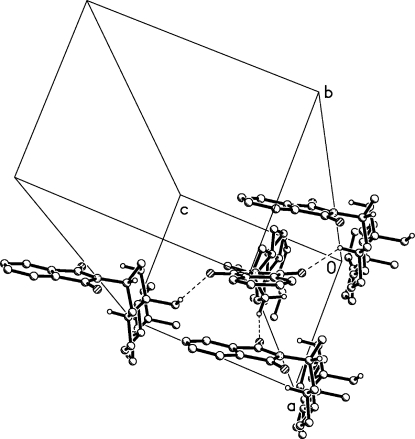
The packing of the title compound, showing the two-dimensional structure, with intermolecular hydrogen bonds (dashed lines); for clarity H atoms not involved in H-bonds have been omitted.

**Fig. 4.**
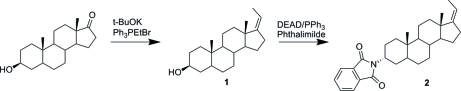
The scheme of synthesis of the title compound.

## supplementary crystallographic information

### Comment

The title compound was synthesized from epiandrosterone which is a pregnane alkaloids isolated from *Pachysandra axillaris*, a Traditional Chinese Medicine (TCM). The pregnane alkaloids from *Pachysandra axillaris* had been reported to be effective as anticancer (Sun *et al.*, 2010).

The title compound, crystallizes with two independent molecules in an asymmetric unit. The cyclohexyl rings (*A*) of the pregnene moiety bonded to the 3α-(1,3-dioxoisoindolin-2-yl)- ring system in both molecules differ in conformations; in one molecule (Fig. 1) it is in a chair conformation while in the other molecule (Fig. 2) it exhibits a half chair conformation. The conformations of the rings B–D in the two molecules are identical with six membered rings (B & C) in chair conformations and the five membered rings in envelope forms in both molecules. In both molecules, the 3α-(1,3-dioxoisoindolin-2-yl)- ring systems are individually planar with rms devtaions 0.0148 and 0.0264 Å. The structure is consolidated by intermolecular hydrogen bonding interactions of the type C—H···O involving carbonyl O atoms and methyl, methylene and methylidyne groups, resulting in a two-dimensional structure (Fig. 3).

### Experimental

The title compound was prepared by following a literature procedure (Batcho *et al.*, 1981). A solution of potassium t-butoxide (t-BuOK) (2.28 g, 20.0 mmol) in 15 ml THF was added to a suspension of Ph_3_PEtBr (7.58 g, 20.0 mmol), in 25 ml dry THF, and was stirred for 1 h at room temperature. The mixture was refluxed for another 4 h after adding epiandrosterone (1.45 g, 5.0 mmol). The residue thus obtained was purified by recrystallization from MeOH to afford 1 (675.8 mg, 45%; Fig. 4). To a solution of 1 (86.9 mg, 0.287 mmol) and triphenylphosphine (85.1 mg, 0.324 mmol) in THF (12 ml), phthalimide (46.5 mg, 0.316 mmol) and diisopropyl azodicarboxylate (63.9 mg, 0.316 mmol) were added, and the mixture was stirred for 18 h at room temperature. The residue was purified by crystallization from MeOH to afford the title compound, 2 (78.0 mg, 63%) as a white solid. The crystals of 2 were obtained by slow evaporation of its solution using a mixed solvent MeOH/CH_2_Cl_2_ (1:1).

### Refinement

H atoms were placed at calculated positions with C—H = 0.95 Å (aryl), 0.98 (methylene), 0.99 (methyl) and 1.00 Å (methyne) and were refined in the riding-model approximation with *U*_iso_ = 1.2–1.5 times *U*_eq_ of the parent atoms. As the structure has no anomalous scatterer, an absolute structure could not be established in this analysis; the Friedel-pairs (3600) of reflections  were merged.

### Crystal data

**Table:**

C_29_H_37_NO_2_	*F*(000) = 936
*M**_r_* = 431.60	*D*_x_ = 1.220 Mg m^−^^3^
Monoclinic, *P*2_1_	Mo *K*α radiation, λ = 0.71073 Å
Hall symbol: P 2yb	Cell parameters from 7024 reflections
*a* = 7.5895 (10) Å	θ = 1.9–28.4°
*b* = 31.355 (3) Å	µ = 0.08 mm^−^^1^
*c* = 9.8912 (12) Å	*T* = 113 K
β = 93.056 (6)°	Prism, colorless
*V* = 2350.4 (5) Å^3^	0.22 × 0.20 × 0.12 mm
*Z* = 4	

### Data collection

**Table:**

Rigaku Saturn724 CCD diffractometer	5642 independent reflections
Radiation source: rotating anode	5296 reflections with *I* > 2σ(*I*)
multilayer	*R*_int_ = 0.052
Detector resolution: 14.22 pixels mm^-1^	θ_max_ = 27.9°, θ_min_ = 2.1°
ω and φ scans	*h* = −9→9
Absorption correction: multi-scan (*CrystalClear*; Rigaku/MSC, 2005)	*k* = −41→35
*T*_min_ = 0.984, *T*_max_ = 0.991	*l* = −12→13
17383 measured reflections	

### Refinement

**Table:**

Refinement on *F*^2^	Primary atom site location: structure-invariant direct methods
Least-squares matrix: full	Secondary atom site location: difference Fourier map
*R*[*F*^2^ > 2σ(*F*^2^)] = 0.039	Hydrogen site location: inferred from neighbouring sites
*wR*(*F*^2^) = 0.096	H-atom parameters constrained
*S* = 1.04	*w* = 1/[σ^2^(*F*_o_^2^) + (0.0522*P*)^2^ + 0.1053*P*] where *P* = (*F*_o_^2^ + 2*F*_c_^2^)/3
5642 reflections	(Δ/σ)_max_ < 0.001
583 parameters	Δρ_max_ = 0.26 e Å^−^^3^
1 restraint	Δρ_min_ = −0.27 e Å^−^^3^

### Special details

**Table:**

Geometry. All e.s.d.'s (except the e.s.d. in the dihedral angle between two l.s. planes) are estimated using the full covariance matrix. The cell e.s.d.'s are taken into account individually in the estimation of e.s.d.'s in distances, angles and torsion angles; correlations between e.s.d.'s in cell parameters are only used when they are defined by crystal symmetry. An approximate (isotropic) treatment of cell e.s.d.'s is used for estimating e.s.d.'s involving l.s. planes.
Refinement. Refinement of *F*^2^ against ALL reflections. The weighted *R*-factor *wR* and goodness of fit *S* are based on *F*^2^, conventional *R*-factors *R* are based on *F*, with *F* set to zero for negative *F*^2^. The threshold expression of *F*^2^ > σ(*F*^2^) is used only for calculating *R*-factors(gt) *etc*. and is not relevant to the choice of reflections for refinement. *R*-factors based on *F*^2^ are statistically about twice as large as those based on *F*, and *R*- factors based on ALL data will be even larger.

### Fractional atomic coordinates and isotropic or equivalent isotropic displacement parameters (Å^2^)

**Table:**

	*x*	*y*	*z*	*U*_iso_*/*U*_eq_	
O1	0.0919 (2)	0.42410 (5)	0.94269 (15)	0.0332 (4)	
O2	−0.0642 (2)	0.30027 (5)	1.15244 (15)	0.0323 (4)	
O3	0.6594 (2)	0.33129 (5)	0.49697 (15)	0.0363 (4)	
O4	0.4062 (2)	0.30622 (5)	0.07334 (14)	0.0282 (3)	
N1	−0.0051 (2)	0.35800 (5)	1.01138 (16)	0.0212 (3)	
N2	0.5259 (2)	0.30729 (5)	0.29473 (16)	0.0209 (3)	
C1	−0.2120 (3)	0.27009 (6)	0.8787 (2)	0.0226 (4)	
H1A	−0.3231	0.2561	0.8459	0.027*	
H1B	−0.1964	0.2647	0.9772	0.027*	
C2	−0.2298 (3)	0.31824 (7)	0.8555 (2)	0.0245 (4)	
H2A	−0.3138	0.3296	0.9197	0.029*	
H2B	−0.2824	0.3229	0.7629	0.029*	
C3	−0.0590 (3)	0.34439 (6)	0.87136 (19)	0.0224 (4)	
H3	−0.0820	0.3712	0.8188	0.027*	
C4	0.0929 (3)	0.32200 (6)	0.80395 (19)	0.0222 (4)	
H4A	0.0725	0.3237	0.7044	0.027*	
H4B	0.2045	0.3371	0.8286	0.027*	
C5	0.1118 (3)	0.27542 (6)	0.84529 (18)	0.0194 (4)	
H5	0.1287	0.2749	0.9462	0.023*	
C6	0.2767 (3)	0.25537 (7)	0.78951 (19)	0.0218 (4)	
H6A	0.2656	0.2555	0.6893	0.026*	
H6B	0.3814	0.2726	0.8183	0.026*	
C7	0.3024 (2)	0.20970 (6)	0.83963 (19)	0.0209 (4)	
H7A	0.3314	0.2101	0.9384	0.025*	
H7B	0.4033	0.1968	0.7951	0.025*	
C8	0.1381 (2)	0.18215 (6)	0.81060 (19)	0.0188 (4)	
H8	0.1181	0.1790	0.7103	0.023*	
C9	−0.0256 (2)	0.20368 (6)	0.86728 (18)	0.0181 (4)	
H9	0.0025	0.2076	0.9664	0.022*	
C10	−0.0571 (3)	0.24924 (6)	0.80799 (18)	0.0193 (4)	
C11	−0.1900 (3)	0.17485 (7)	0.8555 (2)	0.0233 (4)	
H11A	−0.2851	0.1884	0.9052	0.028*	
H11B	−0.2312	0.1730	0.7591	0.028*	
C12	−0.1595 (3)	0.12943 (7)	0.9107 (2)	0.0224 (4)	
H12A	−0.1375	0.1306	1.0102	0.027*	
H12B	−0.2667	0.1120	0.8909	0.027*	
C13	−0.0016 (3)	0.10837 (6)	0.84645 (19)	0.0200 (4)	
C14	0.1594 (2)	0.13797 (6)	0.87296 (18)	0.0192 (4)	
H14	0.1701	0.1425	0.9732	0.023*	
C15	0.3180 (3)	0.11070 (7)	0.8388 (2)	0.0252 (4)	
H15A	0.4285	0.1218	0.8837	0.030*	
H15B	0.3313	0.1095	0.7399	0.030*	
C16	0.2700 (3)	0.06657 (7)	0.8955 (2)	0.0263 (4)	
H16A	0.3051	0.0436	0.8336	0.032*	
H16B	0.3308	0.0619	0.9853	0.032*	
C17	0.0697 (3)	0.06678 (7)	0.90705 (19)	0.0237 (4)	
C18	−0.0176 (3)	0.03466 (7)	0.9622 (2)	0.0291 (5)	
H18	0.0521	0.0113	0.9946	0.035*	
C19	−0.2132 (3)	0.03103 (8)	0.9792 (3)	0.0352 (5)	
H19A	−0.2483	0.0516	1.0474	0.053*	
H19B	−0.2412	0.0021	1.0085	0.053*	
H19C	−0.2772	0.0371	0.8927	0.053*	
C20	−0.0439 (3)	0.10017 (7)	0.6943 (2)	0.0266 (4)	
H20A	0.0583	0.0869	0.6546	0.040*	
H20B	−0.0710	0.1273	0.6486	0.040*	
H20C	−0.1459	0.0811	0.6832	0.040*	
C21	−0.0997 (3)	0.24812 (7)	0.65418 (19)	0.0241 (4)	
H21A	−0.1986	0.2286	0.6341	0.036*	
H21B	0.0040	0.2382	0.6083	0.036*	
H21C	−0.1316	0.2768	0.6222	0.036*	
C22	0.0686 (3)	0.39855 (7)	1.0316 (2)	0.0234 (4)	
C23	−0.0088 (3)	0.33577 (7)	1.13435 (19)	0.0225 (4)	
C24	0.0676 (3)	0.36517 (7)	1.24055 (19)	0.0228 (4)	
C25	0.1115 (3)	0.40315 (7)	1.1793 (2)	0.0246 (4)	
C26	0.1802 (3)	0.43709 (7)	1.2540 (2)	0.0327 (5)	
H26	0.2100	0.4632	1.2120	0.039*	
C27	0.2040 (4)	0.43139 (8)	1.3936 (2)	0.0387 (6)	
H27	0.2503	0.4542	1.4480	0.046*	
C28	0.1616 (3)	0.39320 (9)	1.4547 (2)	0.0361 (5)	
H28	0.1806	0.3902	1.5499	0.043*	
C29	0.0919 (3)	0.35916 (8)	1.3792 (2)	0.0295 (5)	
H29	0.0623	0.3330	1.4207	0.035*	
C30	0.6180 (3)	0.18753 (7)	0.2866 (2)	0.0267 (4)	
H30A	0.7183	0.1847	0.3541	0.032*	
H30B	0.6414	0.1685	0.2097	0.032*	
C31	0.6114 (3)	0.23375 (7)	0.2351 (2)	0.0266 (4)	
H31A	0.7330	0.2448	0.2291	0.032*	
H31B	0.5527	0.2346	0.1433	0.032*	
C32	0.5104 (3)	0.26194 (6)	0.3308 (2)	0.0220 (4)	
H32	0.5656	0.2582	0.4240	0.026*	
C33	0.3173 (3)	0.24763 (7)	0.3335 (2)	0.0226 (4)	
H33A	0.2446	0.2651	0.2685	0.027*	
H33B	0.2748	0.2528	0.4250	0.027*	
C34	0.2920 (3)	0.20021 (6)	0.29740 (19)	0.0194 (4)	
H34	0.2892	0.1982	0.1964	0.023*	
C35	0.1129 (3)	0.18443 (7)	0.3409 (2)	0.0239 (4)	
H35A	0.1073	0.1880	0.4401	0.029*	
H35B	0.0184	0.2021	0.2967	0.029*	
C36	0.0799 (3)	0.13791 (7)	0.3046 (2)	0.0233 (4)	
H36A	0.0666	0.1351	0.2049	0.028*	
H36B	−0.0318	0.1286	0.3425	0.028*	
C37	0.2294 (2)	0.10888 (6)	0.35822 (18)	0.0186 (4)	
H37	0.2339	0.1096	0.4594	0.022*	
C38	0.4080 (3)	0.12490 (6)	0.30987 (19)	0.0192 (4)	
H38	0.3969	0.1247	0.2087	0.023*	
C39	0.4472 (2)	0.17192 (6)	0.35221 (18)	0.0184 (4)	
C40	0.5592 (3)	0.09406 (7)	0.3504 (2)	0.0278 (4)	
H40A	0.6672	0.1036	0.3071	0.033*	
H40B	0.5829	0.0956	0.4497	0.033*	
C41	0.5217 (3)	0.04757 (7)	0.3106 (2)	0.0287 (5)	
H41A	0.6196	0.0293	0.3470	0.034*	
H41B	0.5159	0.0450	0.2107	0.034*	
C42	0.3478 (3)	0.03193 (7)	0.3651 (2)	0.0233 (4)	
C43	0.2021 (3)	0.06299 (6)	0.31204 (18)	0.0209 (4)	
H43	0.2081	0.0634	0.2113	0.025*	
C44	0.0288 (3)	0.04034 (7)	0.3408 (2)	0.0282 (5)	
H44A	−0.0691	0.0504	0.2787	0.034*	
H44B	−0.0030	0.0451	0.4354	0.034*	
C45	0.0704 (3)	−0.00700 (7)	0.3158 (2)	0.0329 (5)	
H45A	0.0266	−0.0249	0.3893	0.039*	
H45B	0.0138	−0.0166	0.2286	0.039*	
C46	0.2709 (3)	−0.01020 (7)	0.3130 (2)	0.0275 (5)	
C47	0.3507 (4)	−0.04530 (8)	0.2710 (2)	0.0346 (5)	
H47	0.2742	−0.0674	0.2378	0.042*	
C48	0.5431 (4)	−0.05482 (8)	0.2688 (3)	0.0404 (6)	
H48A	0.5838	−0.0492	0.1782	0.061*	
H48B	0.5637	−0.0849	0.2919	0.061*	
H48C	0.6082	−0.0367	0.3349	0.061*	
C49	0.3627 (3)	0.02932 (7)	0.5214 (2)	0.0294 (5)	
H49A	0.2481	0.0214	0.5550	0.044*	
H49B	0.3989	0.0571	0.5587	0.044*	
H49C	0.4508	0.0078	0.5496	0.044*	
C50	0.4707 (3)	0.17570 (7)	0.5074 (2)	0.0251 (4)	
H50A	0.4909	0.2056	0.5324	0.038*	
H50B	0.5720	0.1585	0.5401	0.038*	
H50C	0.3640	0.1654	0.5484	0.038*	
C51	0.6074 (3)	0.33777 (7)	0.3812 (2)	0.0241 (4)	
C52	0.4818 (3)	0.32479 (6)	0.16710 (19)	0.0215 (4)	
C53	0.5465 (3)	0.36949 (6)	0.17163 (19)	0.0199 (4)	
C54	0.6196 (3)	0.37753 (6)	0.30028 (19)	0.0225 (4)	
C55	0.6941 (3)	0.41651 (7)	0.3343 (2)	0.0278 (4)	
H55	0.7417	0.4223	0.4233	0.033*	
C56	0.6962 (3)	0.44707 (7)	0.2317 (2)	0.0303 (5)	
H56	0.7488	0.4740	0.2508	0.036*	
C57	0.6231 (3)	0.43903 (7)	0.1023 (2)	0.0282 (5)	
H57	0.6266	0.4605	0.0349	0.034*	
C58	0.5448 (3)	0.39987 (7)	0.0701 (2)	0.0256 (4)	
H58	0.4927	0.3943	−0.0175	0.031*	

### Atomic displacement parameters (Å^2^)

**Table:**

	*U*^11^	*U*^22^	*U*^33^	*U*^12^	*U*^13^	*U*^23^
O1	0.0471 (10)	0.0241 (8)	0.0284 (8)	−0.0045 (7)	0.0028 (7)	0.0046 (6)
O2	0.0435 (9)	0.0258 (8)	0.0274 (7)	−0.0104 (7)	0.0000 (6)	0.0038 (6)
O3	0.0557 (11)	0.0276 (9)	0.0240 (7)	−0.0018 (8)	−0.0127 (7)	0.0044 (6)
O4	0.0325 (8)	0.0286 (8)	0.0228 (7)	−0.0053 (6)	−0.0053 (6)	0.0008 (6)
N1	0.0242 (8)	0.0197 (8)	0.0198 (7)	0.0024 (6)	0.0026 (6)	0.0013 (6)
N2	0.0229 (8)	0.0184 (8)	0.0211 (8)	−0.0003 (7)	−0.0004 (6)	0.0033 (6)
C1	0.0161 (9)	0.0247 (11)	0.0270 (9)	0.0017 (7)	0.0010 (7)	−0.0031 (7)
C2	0.0233 (10)	0.0246 (11)	0.0257 (9)	0.0049 (8)	0.0021 (8)	−0.0024 (7)
C3	0.0258 (10)	0.0201 (10)	0.0211 (9)	0.0035 (8)	0.0006 (8)	0.0012 (7)
C4	0.0238 (10)	0.0222 (10)	0.0208 (9)	−0.0004 (8)	0.0035 (7)	−0.0012 (7)
C5	0.0180 (9)	0.0224 (10)	0.0177 (8)	0.0015 (7)	0.0017 (7)	−0.0021 (7)
C6	0.0194 (9)	0.0253 (10)	0.0212 (9)	−0.0001 (8)	0.0038 (7)	−0.0031 (7)
C7	0.0148 (9)	0.0272 (10)	0.0207 (9)	−0.0001 (7)	0.0015 (7)	−0.0030 (7)
C8	0.0163 (9)	0.0226 (10)	0.0174 (8)	0.0008 (7)	0.0009 (7)	−0.0031 (7)
C9	0.0149 (9)	0.0220 (10)	0.0176 (8)	0.0013 (7)	0.0016 (7)	−0.0019 (7)
C10	0.0190 (9)	0.0212 (10)	0.0176 (8)	0.0021 (7)	−0.0001 (7)	−0.0013 (7)
C11	0.0171 (9)	0.0238 (10)	0.0288 (10)	0.0010 (8)	0.0004 (7)	−0.0032 (8)
C12	0.0170 (9)	0.0250 (10)	0.0253 (9)	0.0000 (8)	0.0020 (7)	0.0002 (7)
C13	0.0191 (9)	0.0225 (10)	0.0184 (8)	0.0003 (8)	0.0018 (7)	−0.0014 (7)
C14	0.0168 (9)	0.0227 (10)	0.0181 (8)	0.0033 (7)	0.0014 (7)	−0.0008 (7)
C15	0.0206 (9)	0.0247 (10)	0.0307 (10)	0.0052 (8)	0.0052 (8)	−0.0007 (8)
C16	0.0254 (11)	0.0265 (11)	0.0277 (10)	0.0062 (8)	0.0069 (8)	0.0031 (8)
C17	0.0255 (10)	0.0237 (10)	0.0222 (9)	0.0023 (8)	0.0038 (8)	−0.0028 (7)
C18	0.0324 (12)	0.0257 (11)	0.0299 (10)	0.0039 (9)	0.0081 (9)	0.0009 (8)
C19	0.0364 (13)	0.0272 (12)	0.0432 (13)	−0.0030 (10)	0.0126 (10)	0.0018 (9)
C20	0.0301 (11)	0.0282 (11)	0.0211 (9)	−0.0044 (8)	−0.0017 (8)	−0.0038 (7)
C21	0.0280 (11)	0.0249 (10)	0.0190 (8)	0.0017 (8)	−0.0030 (8)	−0.0016 (7)
C22	0.0269 (10)	0.0203 (10)	0.0233 (9)	0.0032 (8)	0.0040 (8)	0.0011 (7)
C23	0.0251 (10)	0.0218 (10)	0.0208 (9)	0.0019 (8)	0.0038 (7)	0.0017 (7)
C24	0.0222 (10)	0.0235 (10)	0.0229 (9)	0.0008 (8)	0.0038 (7)	−0.0011 (7)
C25	0.0253 (10)	0.0231 (10)	0.0260 (10)	0.0011 (8)	0.0056 (8)	−0.0007 (8)
C26	0.0400 (13)	0.0256 (12)	0.0329 (11)	−0.0053 (10)	0.0055 (10)	−0.0038 (8)
C27	0.0498 (15)	0.0345 (13)	0.0316 (12)	−0.0098 (11)	0.0010 (10)	−0.0110 (9)
C28	0.0423 (14)	0.0419 (14)	0.0240 (10)	−0.0056 (11)	−0.0005 (9)	−0.0034 (9)
C29	0.0341 (12)	0.0315 (12)	0.0232 (10)	−0.0018 (9)	0.0034 (8)	0.0023 (8)
C30	0.0186 (10)	0.0220 (11)	0.0401 (12)	−0.0002 (8)	0.0071 (9)	0.0036 (8)
C31	0.0219 (10)	0.0211 (10)	0.0376 (11)	−0.0012 (8)	0.0098 (9)	0.0030 (8)
C32	0.0244 (10)	0.0167 (9)	0.0249 (9)	−0.0010 (8)	0.0000 (8)	0.0047 (7)
C33	0.0212 (10)	0.0231 (10)	0.0238 (9)	0.0030 (8)	0.0046 (7)	0.0033 (7)
C34	0.0179 (9)	0.0211 (10)	0.0191 (8)	−0.0001 (7)	0.0012 (7)	0.0019 (7)
C35	0.0178 (9)	0.0231 (11)	0.0310 (10)	0.0017 (8)	0.0041 (8)	0.0011 (8)
C36	0.0160 (9)	0.0252 (11)	0.0288 (10)	−0.0026 (8)	0.0016 (7)	0.0009 (8)
C37	0.0173 (9)	0.0204 (9)	0.0183 (8)	−0.0020 (7)	0.0027 (7)	0.0001 (7)
C38	0.0174 (9)	0.0196 (9)	0.0211 (8)	−0.0013 (7)	0.0050 (7)	0.0006 (7)
C39	0.0163 (9)	0.0182 (9)	0.0209 (8)	−0.0007 (7)	0.0022 (7)	0.0008 (7)
C40	0.0198 (10)	0.0204 (10)	0.0434 (12)	0.0008 (8)	0.0042 (9)	0.0048 (9)
C41	0.0260 (11)	0.0217 (11)	0.0395 (12)	0.0014 (8)	0.0105 (9)	0.0008 (8)
C42	0.0275 (10)	0.0193 (10)	0.0238 (9)	−0.0016 (8)	0.0069 (8)	−0.0012 (7)
C43	0.0228 (10)	0.0214 (10)	0.0191 (8)	−0.0044 (8)	0.0052 (7)	−0.0024 (7)
C44	0.0254 (11)	0.0276 (11)	0.0324 (11)	−0.0084 (8)	0.0078 (8)	−0.0069 (8)
C45	0.0370 (13)	0.0246 (11)	0.0383 (12)	−0.0105 (9)	0.0132 (10)	−0.0057 (9)
C46	0.0373 (12)	0.0219 (10)	0.0241 (10)	−0.0054 (9)	0.0092 (9)	−0.0010 (8)
C47	0.0450 (14)	0.0272 (12)	0.0318 (11)	−0.0028 (10)	0.0030 (10)	−0.0057 (9)
C48	0.0478 (15)	0.0281 (13)	0.0442 (13)	0.0077 (11)	−0.0080 (11)	−0.0132 (10)
C49	0.0389 (12)	0.0245 (11)	0.0248 (10)	0.0028 (9)	0.0027 (9)	0.0015 (8)
C50	0.0298 (11)	0.0212 (10)	0.0235 (9)	−0.0036 (8)	−0.0040 (8)	0.0029 (7)
C51	0.0283 (11)	0.0215 (10)	0.0224 (9)	0.0000 (8)	−0.0013 (8)	0.0004 (7)
C52	0.0203 (10)	0.0227 (10)	0.0215 (9)	0.0007 (7)	0.0008 (7)	0.0037 (7)
C53	0.0197 (9)	0.0181 (9)	0.0221 (9)	0.0026 (7)	0.0023 (7)	−0.0001 (7)
C54	0.0242 (10)	0.0213 (10)	0.0219 (9)	0.0025 (8)	0.0015 (7)	0.0002 (7)
C55	0.0345 (12)	0.0207 (10)	0.0280 (10)	0.0008 (9)	−0.0001 (8)	−0.0031 (8)
C56	0.0376 (13)	0.0192 (10)	0.0343 (11)	−0.0020 (9)	0.0048 (9)	−0.0035 (8)
C57	0.0392 (13)	0.0189 (10)	0.0274 (10)	0.0041 (9)	0.0085 (9)	0.0039 (7)
C58	0.0324 (11)	0.0231 (10)	0.0215 (9)	0.0028 (8)	0.0043 (8)	0.0018 (7)

### Geometric parameters (Å, °)

**Table:**

O1—C22	1.210 (2)	C27—C28	1.387 (4)
O2—C23	1.207 (3)	C27—H27	0.9500
O3—C51	1.208 (2)	C28—C29	1.391 (3)
O4—C52	1.213 (2)	C28—H28	0.9500
N1—C22	1.399 (3)	C29—H29	0.9500
N1—C23	1.403 (2)	C30—C31	1.536 (3)
N1—C3	1.486 (2)	C30—C39	1.559 (3)
N2—C52	1.401 (2)	C30—H30A	0.9900
N2—C51	1.404 (3)	C30—H30B	0.9900
N2—C32	1.472 (2)	C31—C32	1.531 (3)
C1—C2	1.532 (3)	C31—H31A	0.9900
C1—C10	1.544 (3)	C31—H31B	0.9900
C1—H1A	0.9900	C32—C33	1.534 (3)
C1—H1B	0.9900	C32—H32	1.0000
C2—C3	1.534 (3)	C33—C34	1.539 (3)
C2—H2A	0.9900	C33—H33A	0.9900
C2—H2B	0.9900	C33—H33B	0.9900
C3—C4	1.532 (3)	C34—C35	1.530 (3)
C3—H3	1.0000	C34—C39	1.549 (3)
C4—C5	1.521 (3)	C34—H34	1.0000
C4—H4A	0.9900	C35—C36	1.520 (3)
C4—H4B	0.9900	C35—H35A	0.9900
C5—C6	1.529 (3)	C35—H35B	0.9900
C5—C10	1.550 (3)	C36—C37	1.527 (3)
C5—H5	1.0000	C36—H36A	0.9900
C6—C7	1.525 (3)	C36—H36B	0.9900
C6—H6A	0.9900	C37—C43	1.521 (3)
C6—H6B	0.9900	C37—C38	1.545 (2)
C7—C8	1.531 (3)	C37—H37	1.0000
C7—H7A	0.9900	C38—C40	1.537 (3)
C7—H7B	0.9900	C38—C39	1.557 (3)
C8—C14	1.521 (3)	C38—H38	1.0000
C8—C9	1.546 (2)	C39—C50	1.540 (3)
C8—H8	1.0000	C40—C41	1.533 (3)
C9—C11	1.540 (3)	C40—H40A	0.9900
C9—C10	1.558 (3)	C40—H40B	0.9900
C9—H9	1.0000	C41—C42	1.533 (3)
C10—C21	1.539 (2)	C41—H41A	0.9900
C11—C12	1.538 (3)	C41—H41B	0.9900
C11—H11A	0.9900	C42—C46	1.523 (3)
C11—H11B	0.9900	C42—C43	1.544 (3)
C12—C13	1.535 (3)	C42—C49	1.547 (3)
C12—H12A	0.9900	C43—C44	1.534 (3)
C12—H12B	0.9900	C43—H43	1.0000
C13—C17	1.523 (3)	C44—C45	1.540 (3)
C13—C20	1.543 (3)	C44—H44A	0.9900
C13—C14	1.546 (3)	C44—H44B	0.9900
C14—C15	1.529 (3)	C45—C46	1.527 (3)
C14—H14	1.0000	C45—H45A	0.9900
C15—C16	1.544 (3)	C45—H45B	0.9900
C15—H15A	0.9900	C46—C47	1.333 (3)
C15—H15B	0.9900	C47—C48	1.492 (4)
C16—C17	1.530 (3)	C47—H47	0.9500
C16—H16A	0.9900	C48—H48A	0.9800
C16—H16B	0.9900	C48—H48B	0.9800
C17—C18	1.338 (3)	C48—H48C	0.9800
C18—C19	1.507 (3)	C49—H49A	0.9800
C18—H18	0.9500	C49—H49B	0.9800
C19—H19A	0.9800	C49—H49C	0.9800
C19—H19B	0.9800	C50—H50A	0.9800
C19—H19C	0.9800	C50—H50B	0.9800
C20—H20A	0.9800	C50—H50C	0.9800
C20—H20B	0.9800	C51—C54	1.487 (3)
C20—H20C	0.9800	C52—C53	1.485 (3)
C21—H21A	0.9800	C53—C58	1.384 (3)
C21—H21B	0.9800	C53—C54	1.384 (3)
C21—H21C	0.9800	C54—C55	1.381 (3)
C22—C25	1.487 (3)	C55—C56	1.397 (3)
C23—C24	1.491 (3)	C55—H55	0.9500
C24—C25	1.384 (3)	C56—C57	1.391 (3)
C24—C29	1.387 (3)	C56—H56	0.9500
C25—C26	1.381 (3)	C57—C58	1.393 (3)
C26—C27	1.394 (3)	C57—H57	0.9500
C26—H26	0.9500	C58—H58	0.9500
			
C22—N1—C23	110.72 (17)	C24—C29—C28	116.9 (2)
C22—N1—C3	118.85 (16)	C24—C29—H29	121.5
C23—N1—C3	130.38 (17)	C28—C29—H29	121.5
C52—N2—C51	111.04 (16)	C31—C30—C39	114.93 (17)
C52—N2—C32	125.32 (17)	C31—C30—H30A	108.5
C51—N2—C32	123.24 (16)	C39—C30—H30A	108.5
C2—C1—C10	114.37 (17)	C31—C30—H30B	108.5
C2—C1—H1A	108.7	C39—C30—H30B	108.5
C10—C1—H1A	108.7	H30A—C30—H30B	107.5
C2—C1—H1B	108.7	C32—C31—C30	110.37 (16)
C10—C1—H1B	108.7	C32—C31—H31A	109.6
H1A—C1—H1B	107.6	C30—C31—H31A	109.6
C1—C2—C3	116.35 (17)	C32—C31—H31B	109.6
C1—C2—H2A	108.2	C30—C31—H31B	109.6
C3—C2—H2A	108.2	H31A—C31—H31B	108.1
C1—C2—H2B	108.2	N2—C32—C31	111.04 (16)
C3—C2—H2B	108.2	N2—C32—C33	112.05 (17)
H2A—C2—H2B	107.4	C31—C32—C33	110.65 (17)
N1—C3—C4	111.16 (16)	N2—C32—H32	107.6
N1—C3—C2	115.98 (16)	C31—C32—H32	107.6
C4—C3—C2	111.20 (17)	C33—C32—H32	107.6
N1—C3—H3	105.9	C32—C33—C34	112.69 (16)
C4—C3—H3	105.9	C32—C33—H33A	109.1
C2—C3—H3	105.9	C34—C33—H33A	109.1
C5—C4—C3	112.65 (16)	C32—C33—H33B	109.1
C5—C4—H4A	109.1	C34—C33—H33B	109.1
C3—C4—H4A	109.1	H33A—C33—H33B	107.8
C5—C4—H4B	109.1	C35—C34—C33	110.41 (16)
C3—C4—H4B	109.1	C35—C34—C39	112.75 (15)
H4A—C4—H4B	107.8	C33—C34—C39	112.90 (16)
C4—C5—C6	111.40 (16)	C35—C34—H34	106.8
C4—C5—C10	112.30 (16)	C33—C34—H34	106.8
C6—C5—C10	112.24 (16)	C39—C34—H34	106.8
C4—C5—H5	106.8	C36—C35—C34	112.41 (16)
C6—C5—H5	106.8	C36—C35—H35A	109.1
C10—C5—H5	106.8	C34—C35—H35A	109.1
C7—C6—C5	111.21 (15)	C36—C35—H35B	109.1
C7—C6—H6A	109.4	C34—C35—H35B	109.1
C5—C6—H6A	109.4	H35A—C35—H35B	107.9
C7—C6—H6B	109.4	C35—C36—C37	112.34 (17)
C5—C6—H6B	109.4	C35—C36—H36A	109.1
H6A—C6—H6B	108.0	C37—C36—H36A	109.1
C6—C7—C8	112.34 (16)	C35—C36—H36B	109.1
C6—C7—H7A	109.1	C37—C36—H36B	109.1
C8—C7—H7A	109.1	H36A—C36—H36B	107.9
C6—C7—H7B	109.1	C43—C37—C36	111.88 (16)
C8—C7—H7B	109.1	C43—C37—C38	108.82 (15)
H7A—C7—H7B	107.9	C36—C37—C38	110.27 (15)
C14—C8—C7	111.70 (16)	C43—C37—H37	108.6
C14—C8—C9	108.70 (15)	C36—C37—H37	108.6
C7—C8—C9	110.37 (15)	C38—C37—H37	108.6
C14—C8—H8	108.7	C40—C38—C37	111.70 (16)
C7—C8—H8	108.7	C40—C38—C39	113.34 (17)
C9—C8—H8	108.7	C37—C38—C39	112.50 (15)
C11—C9—C8	112.36 (16)	C40—C38—H38	106.2
C11—C9—C10	113.73 (16)	C37—C38—H38	106.2
C8—C9—C10	111.96 (15)	C39—C38—H38	106.2
C11—C9—H9	106.0	C50—C39—C34	110.48 (15)
C8—C9—H9	106.0	C50—C39—C38	110.56 (15)
C10—C9—H9	106.0	C34—C39—C38	108.50 (15)
C21—C10—C1	109.42 (16)	C50—C39—C30	109.65 (16)
C21—C10—C5	112.02 (15)	C34—C39—C30	107.93 (15)
C1—C10—C5	107.91 (15)	C38—C39—C30	109.66 (15)
C21—C10—C9	111.78 (16)	C41—C40—C38	113.94 (18)
C1—C10—C9	108.84 (15)	C41—C40—H40A	108.8
C5—C10—C9	106.73 (15)	C38—C40—H40A	108.8
C12—C11—C9	114.24 (16)	C41—C40—H40B	108.8
C12—C11—H11A	108.7	C38—C40—H40B	108.8
C9—C11—H11A	108.7	H40A—C40—H40B	107.7
C12—C11—H11B	108.7	C40—C41—C42	111.44 (17)
C9—C11—H11B	108.7	C40—C41—H41A	109.3
H11A—C11—H11B	107.6	C42—C41—H41A	109.3
C13—C12—C11	110.97 (16)	C40—C41—H41B	109.3
C13—C12—H12A	109.4	C42—C41—H41B	109.3
C11—C12—H12A	109.4	H41A—C41—H41B	108.0
C13—C12—H12B	109.4	C46—C42—C41	118.69 (17)
C11—C12—H12B	109.4	C46—C42—C43	100.25 (17)
H12A—C12—H12B	108.0	C41—C42—C43	107.07 (16)
C17—C13—C12	118.33 (16)	C46—C42—C49	107.39 (16)
C17—C13—C20	106.99 (16)	C41—C42—C49	110.45 (19)
C12—C13—C20	110.40 (17)	C43—C42—C49	112.71 (16)
C17—C13—C14	100.73 (15)	C37—C43—C44	119.13 (16)
C12—C13—C14	107.40 (16)	C37—C43—C42	114.27 (16)
C20—C13—C14	112.77 (15)	C44—C43—C42	104.49 (17)
C8—C14—C15	119.19 (16)	C37—C43—H43	106.0
C8—C14—C13	114.32 (16)	C44—C43—H43	106.0
C15—C14—C13	104.56 (16)	C42—C43—H43	106.0
C8—C14—H14	105.9	C43—C44—C45	103.37 (17)
C15—C14—H14	105.9	C43—C44—H44A	111.1
C13—C14—H14	105.9	C45—C44—H44A	111.1
C14—C15—C16	102.55 (16)	C43—C44—H44B	111.1
C14—C15—H15A	111.3	C45—C44—H44B	111.1
C16—C15—H15A	111.3	H44A—C44—H44B	109.1
C14—C15—H15B	111.3	C46—C45—C44	106.23 (17)
C16—C15—H15B	111.3	C46—C45—H45A	110.5
H15A—C15—H15B	109.2	C44—C45—H45A	110.5
C17—C16—C15	106.14 (17)	C46—C45—H45B	110.5
C17—C16—H16A	110.5	C44—C45—H45B	110.5
C15—C16—H16A	110.5	H45A—C45—H45B	108.7
C17—C16—H16B	110.5	C47—C46—C42	130.5 (2)
C15—C16—H16B	110.5	C47—C46—C45	122.0 (2)
H16A—C16—H16B	108.7	C42—C46—C45	107.50 (17)
C18—C17—C13	129.17 (19)	C46—C47—C48	129.1 (2)
C18—C17—C16	122.9 (2)	C46—C47—H47	115.4
C13—C17—C16	107.93 (16)	C48—C47—H47	115.4
C17—C18—C19	127.9 (2)	C47—C48—H48A	109.5
C17—C18—H18	116.0	C47—C48—H48B	109.5
C19—C18—H18	116.0	H48A—C48—H48B	109.5
C18—C19—H19A	109.5	C47—C48—H48C	109.5
C18—C19—H19B	109.5	H48A—C48—H48C	109.5
H19A—C19—H19B	109.5	H48B—C48—H48C	109.5
C18—C19—H19C	109.5	C42—C49—H49A	109.5
H19A—C19—H19C	109.5	C42—C49—H49B	109.5
H19B—C19—H19C	109.5	H49A—C49—H49B	109.5
C13—C20—H20A	109.5	C42—C49—H49C	109.5
C13—C20—H20B	109.5	H49A—C49—H49C	109.5
H20A—C20—H20B	109.5	H49B—C49—H49C	109.5
C13—C20—H20C	109.5	C39—C50—H50A	109.5
H20A—C20—H20C	109.5	C39—C50—H50B	109.5
H20B—C20—H20C	109.5	H50A—C50—H50B	109.5
C10—C21—H21A	109.5	C39—C50—H50C	109.5
C10—C21—H21B	109.5	H50A—C50—H50C	109.5
H21A—C21—H21B	109.5	H50B—C50—H50C	109.5
C10—C21—H21C	109.5	O3—C51—N2	125.04 (19)
H21A—C21—H21C	109.5	O3—C51—C54	128.65 (19)
H21B—C21—H21C	109.5	N2—C51—C54	106.28 (16)
O1—C22—N1	124.81 (19)	O4—C52—N2	125.56 (19)
O1—C22—C25	128.1 (2)	O4—C52—C53	128.19 (18)
N1—C22—C25	107.07 (16)	N2—C52—C53	106.25 (17)
O2—C23—N1	127.59 (19)	C58—C53—C54	121.97 (19)
O2—C23—C24	126.24 (18)	C58—C53—C52	129.63 (19)
N1—C23—C24	106.16 (17)	C54—C53—C52	108.38 (17)
C25—C24—C29	121.8 (2)	C55—C54—C53	121.63 (18)
C25—C24—C23	108.49 (17)	C55—C54—C51	130.32 (18)
C29—C24—C23	129.69 (19)	C53—C54—C51	107.99 (17)
C26—C25—C24	121.4 (2)	C54—C55—C56	116.76 (19)
C26—C25—C22	131.0 (2)	C54—C55—H55	121.6
C24—C25—C22	107.53 (18)	C56—C55—H55	121.6
C25—C26—C27	117.1 (2)	C57—C56—C55	121.7 (2)
C25—C26—H26	121.4	C57—C56—H56	119.2
C27—C26—H26	121.4	C55—C56—H56	119.2
C28—C27—C26	121.4 (2)	C56—C57—C58	120.95 (19)
C28—C27—H27	119.3	C56—C57—H57	119.5
C26—C27—H27	119.3	C58—C57—H57	119.5
C27—C28—C29	121.3 (2)	C53—C58—C57	116.98 (19)
C27—C28—H28	119.4	C53—C58—H58	121.5
C29—C28—H28	119.4	C57—C58—H58	121.5
			
C10—C1—C2—C3	−45.7 (2)	C39—C30—C31—C32	37.1 (3)
C22—N1—C3—C4	90.0 (2)	C52—N2—C32—C31	55.2 (3)
C23—N1—C3—C4	−87.3 (2)	C51—N2—C32—C31	−117.0 (2)
C22—N1—C3—C2	−141.66 (18)	C52—N2—C32—C33	−69.1 (2)
C23—N1—C3—C2	41.1 (3)	C51—N2—C32—C33	118.7 (2)
C1—C2—C3—N1	−85.1 (2)	C30—C31—C32—N2	171.34 (17)
C1—C2—C3—C4	43.2 (2)	C30—C31—C32—C33	−63.6 (2)
N1—C3—C4—C5	81.2 (2)	N2—C32—C33—C34	150.33 (16)
C2—C3—C4—C5	−49.6 (2)	C31—C32—C33—C34	25.8 (2)
C3—C4—C5—C6	−173.49 (16)	C32—C33—C34—C35	163.46 (16)
C3—C4—C5—C10	59.6 (2)	C32—C33—C34—C39	36.2 (2)
C4—C5—C6—C7	175.86 (16)	C33—C34—C35—C36	178.74 (16)
C10—C5—C6—C7	−57.2 (2)	C39—C34—C35—C36	−53.9 (2)
C5—C6—C7—C8	53.6 (2)	C34—C35—C36—C37	53.5 (2)
C6—C7—C8—C14	−174.54 (14)	C35—C36—C37—C43	−175.55 (15)
C6—C7—C8—C9	−53.5 (2)	C35—C36—C37—C38	−54.3 (2)
C14—C8—C9—C11	−50.5 (2)	C43—C37—C38—C40	−51.4 (2)
C7—C8—C9—C11	−173.29 (16)	C36—C37—C38—C40	−174.50 (16)
C14—C8—C9—C10	−179.90 (15)	C43—C37—C38—C39	179.77 (15)
C7—C8—C9—C10	57.3 (2)	C36—C37—C38—C39	56.7 (2)
C2—C1—C10—C21	−71.3 (2)	C35—C34—C39—C50	−67.3 (2)
C2—C1—C10—C5	50.8 (2)	C33—C34—C39—C50	58.7 (2)
C2—C1—C10—C9	166.25 (16)	C35—C34—C39—C38	54.08 (19)
C4—C5—C10—C21	62.4 (2)	C33—C34—C39—C38	−179.93 (14)
C6—C5—C10—C21	−64.1 (2)	C35—C34—C39—C30	172.84 (16)
C4—C5—C10—C1	−58.15 (19)	C33—C34—C39—C30	−61.2 (2)
C6—C5—C10—C1	175.42 (16)	C40—C38—C39—C50	−62.6 (2)
C4—C5—C10—C9	−174.99 (14)	C37—C38—C39—C50	65.3 (2)
C6—C5—C10—C9	58.58 (19)	C40—C38—C39—C34	176.08 (15)
C11—C9—C10—C21	−64.7 (2)	C37—C38—C39—C34	−55.99 (19)
C8—C9—C10—C21	64.1 (2)	C40—C38—C39—C30	58.4 (2)
C11—C9—C10—C1	56.3 (2)	C37—C38—C39—C30	−173.65 (16)
C8—C9—C10—C1	−174.97 (15)	C31—C30—C39—C50	−97.8 (2)
C11—C9—C10—C5	172.55 (14)	C31—C30—C39—C34	22.6 (2)
C8—C9—C10—C5	−58.75 (18)	C31—C30—C39—C38	140.61 (18)
C8—C9—C11—C12	50.1 (2)	C37—C38—C40—C41	51.1 (2)
C10—C9—C11—C12	178.62 (15)	C39—C38—C40—C41	179.42 (16)
C9—C11—C12—C13	−53.2 (2)	C38—C40—C41—C42	−54.0 (2)
C11—C12—C13—C17	168.86 (17)	C40—C41—C42—C46	168.13 (19)
C11—C12—C13—C20	−67.4 (2)	C40—C41—C42—C43	55.8 (2)
C11—C12—C13—C14	55.9 (2)	C40—C41—C42—C49	−67.3 (2)
C7—C8—C14—C15	−54.9 (2)	C36—C37—C43—C44	−54.6 (2)
C9—C8—C14—C15	−176.95 (17)	C38—C37—C43—C44	−176.72 (17)
C7—C8—C14—C13	−179.57 (14)	C36—C37—C43—C42	−179.08 (15)
C9—C8—C14—C13	58.41 (19)	C38—C37—C43—C42	58.84 (19)
C17—C13—C14—C8	174.11 (14)	C46—C42—C43—C37	174.66 (15)
C12—C13—C14—C8	−61.45 (19)	C41—C42—C43—C37	−60.9 (2)
C20—C13—C14—C8	60.4 (2)	C49—C42—C43—C37	60.8 (2)
C17—C13—C14—C15	42.02 (17)	C46—C42—C43—C44	42.74 (17)
C12—C13—C14—C15	166.46 (15)	C41—C42—C43—C44	167.20 (16)
C20—C13—C14—C15	−71.7 (2)	C49—C42—C43—C44	−71.1 (2)
C8—C14—C15—C16	−168.54 (17)	C37—C43—C44—C45	−165.23 (18)
C13—C14—C15—C16	−39.30 (19)	C42—C43—C44—C45	−36.2 (2)
C14—C15—C16—C17	20.9 (2)	C43—C44—C45—C46	15.0 (2)
C12—C13—C17—C18	34.9 (3)	C41—C42—C46—C47	31.1 (3)
C20—C13—C17—C18	−90.4 (2)	C43—C42—C46—C47	147.1 (2)
C14—C13—C17—C18	151.6 (2)	C49—C42—C46—C47	−95.0 (3)
C12—C13—C17—C16	−145.12 (17)	C41—C42—C46—C45	−149.3 (2)
C20—C13—C17—C16	89.52 (19)	C43—C42—C46—C45	−33.23 (19)
C14—C13—C17—C16	−28.51 (18)	C49—C42—C46—C45	84.7 (2)
C15—C16—C17—C18	−174.9 (2)	C44—C45—C46—C47	−168.5 (2)
C15—C16—C17—C13	5.1 (2)	C44—C45—C46—C42	11.8 (2)
C13—C17—C18—C19	0.0 (4)	C42—C46—C47—C48	3.4 (4)
C16—C17—C18—C19	−179.9 (2)	C45—C46—C47—C48	−176.2 (2)
C23—N1—C22—O1	178.1 (2)	C52—N2—C51—O3	−179.8 (2)
C3—N1—C22—O1	0.3 (3)	C32—N2—C51—O3	−6.6 (3)
C23—N1—C22—C25	−1.5 (2)	C52—N2—C51—C54	−1.4 (2)
C3—N1—C22—C25	−179.25 (16)	C32—N2—C51—C54	171.68 (17)
C22—N1—C23—O2	179.7 (2)	C51—N2—C52—O4	−178.0 (2)
C3—N1—C23—O2	−2.8 (4)	C32—N2—C52—O4	9.0 (3)
C22—N1—C23—C24	0.6 (2)	C51—N2—C52—C53	2.2 (2)
C3—N1—C23—C24	178.00 (18)	C32—N2—C52—C53	−170.77 (17)
O2—C23—C24—C25	−178.5 (2)	O4—C52—C53—C58	−3.6 (4)
N1—C23—C24—C25	0.6 (2)	N2—C52—C53—C58	176.2 (2)
O2—C23—C24—C29	−0.2 (4)	O4—C52—C53—C54	178.1 (2)
N1—C23—C24—C29	179.0 (2)	N2—C52—C53—C54	−2.1 (2)
C29—C24—C25—C26	−0.6 (3)	C58—C53—C54—C55	0.3 (3)
C23—C24—C25—C26	177.9 (2)	C52—C53—C54—C55	178.75 (19)
C29—C24—C25—C22	−179.99 (19)	C58—C53—C54—C51	−177.17 (19)
C23—C24—C25—C22	−1.5 (2)	C52—C53—C54—C51	1.3 (2)
O1—C22—C25—C26	3.0 (4)	O3—C51—C54—C55	1.1 (4)
N1—C22—C25—C26	−177.5 (2)	N2—C51—C54—C55	−177.2 (2)
O1—C22—C25—C24	−177.7 (2)	O3—C51—C54—C53	178.3 (2)
N1—C22—C25—C24	1.8 (2)	N2—C51—C54—C53	0.1 (2)
C24—C25—C26—C27	0.2 (3)	C53—C54—C55—C56	−1.6 (3)
C22—C25—C26—C27	179.4 (2)	C51—C54—C55—C56	175.3 (2)
C25—C26—C27—C28	0.4 (4)	C54—C55—C56—C57	1.4 (3)
C26—C27—C28—C29	−0.6 (4)	C55—C56—C57—C58	−0.1 (3)
C25—C24—C29—C28	0.4 (3)	C54—C53—C58—C57	1.0 (3)
C23—C24—C29—C28	−177.8 (2)	C52—C53—C58—C57	−177.01 (19)
C27—C28—C29—C24	0.2 (4)	C56—C57—C58—C53	−1.2 (3)

### Hydrogen-bond geometry (Å, °)

**Table:**

*D*—H···*A*	*D*—H	H···*A*	*D*···*A*	*D*—H···*A*
C1—H1B···O2	0.99	2.25	3.027 (3)	134
C3—H3···O1	1.00	2.41	2.823 (3)	104
C5—H5···O4^i^	1.00	2.59	3.237 (3)	122
C21—H21C···O3^ii^	0.98	2.60	3.501 (3)	153
C31—H31A···O2^iii^	0.99	2.47	3.361 (3)	150
C31—H31B···O4	0.99	2.58	3.144 (3)	116
C32—H32···O3	1.00	2.49	2.917 (3)	105

Symmetry codes: (i) *x*, *y*, *z*+1; (ii) *x*−1, *y*, *z*; (iii) *x*+1, *y*, *z*−1.
